# Tarnished Plant Bug (Heteroptera: Miridae) Behavioral Responses to Chemical Insecticides

**DOI:** 10.3390/insects12121072

**Published:** 2021-11-30

**Authors:** Scott H. Graham, Angus L. Catchot, Jeffrey Gore, Donald R. Cook, Darrin Dodds

**Affiliations:** 1 Department of Entomology and Plant Pathology, Auburn University, Auburn, AL 36849, USA; 2 Department of Biochemistry, Molecular Biology, Entomology and Plant Pathology, Mississippi State University, Mississippi State, MS 39762, USA; acatchot@ext.msstate.edu; 3 Delta Research and Extension Center, Mississippi State University, Stoneville, MS 38776, USA; JGore@drec.msstate.edu (J.G.); dcook@drec.msstate.edu (D.R.C.); 4 Department of Plant and Soil Sciences, Mississippi State University, Mississippi State, MS 39762, USA; dmd72@msstate.edu

**Keywords:** behavior, insecticides, tarnished plant bug

## Abstract

**Simple Summary:**

The tarnished plant bug, *Lygus lineolaris* (L.), is a pernicious pest of cotton across the Mid-South and Southeastern US Cotton Belt. In order to manage this pest, numerous insecticide applications are required annually. This has led to widespread resistance and increased cost of control. We tested the behavioral response of tarnished plant bug against various classes of chemistry used for control. The insects avoided certain chemicals and were slightly attracted to others. These findings help us understand the role of tarnished plant bug behavior on field control failures, insecticide resistance, and insecticide resistance management.

**Abstract:**

The tarnished plant bug (*Lygus lineolaris* Palisot de Beauvois) is the dominant insect pest of cotton (*Gossypium hirsutum* L.) in the Mid-South Cotton Belt. This is partly due to the fact that this pest has developed resistance to most insecticides used for control. Laboratory experiments were conducted during 2014 and 2015 to study the behavioral response of tarnished plant bug nymphs to several classes of insecticides. Twenty third-instar nymphs were placed in individual dishes divided into four quadrants with five green bean pieces in each quadrant (10 treated and 10 untreated green beans in each dish). Dishes were checked at 1, 4, 8, and 24 h. Tarnished plant bug nymphs appeared to avoid green beans treated with IGR, pyrethroid, organophosphate, or carbamate insecticides, while there appeared to be an attraction to green bean pieces treated with sulfoxamine and pyridine carboxamide insecticides. No relationship was observed with neonicotinoid insecticides within 24 h.

## 1. Introduction

The tarnished plant bug *Lygus lineolaris* (Palisot de Beauvois) is an important insect pest of cotton in the Mid-South of the US. Prior to the eradication of the boll weevil (*Anthonomas grandis* (Boheman)) and the introduction of *Bacillus thuringiensis* ((Berliner) (Bt)) crops to manage bollworm (*Helicoverpa zea* (Boddie)) and tobacco budworm (*Heliothis virescens* (F.)), the pest status of tarnished plant bug was less than it is now [1]. Insecticide applications targeting the boll weevil, bollworm, and tobacco budworm provided coincidental control of tarnished plant bug and may be an important cause for resistance to organophosphates and pyrethroids in tarnished plant bug [2]. The first documentation of insecticide resistance in the tarnished plant bug was to methyl parathion in populations from the Mississippi Delta during the late 1970s [3]. Subsequently, Snodgrass [4] reported tolerance to dimethoate in the same region. By the mid-1990s, resistance to pyrethroid, organophosphate, and cyclodiene insecticides was reported in the Mississippi Delta [5]. Resistance to pyrethroids has been reported in most counties along the Mississippi River in Arkansas, Louisiana, and Mississippi [2,6,7,8]. More recently, widespread resistance to acephate has been reported in the region [9,10]. Despite extensive resistance, insecticides remain an important component of tarnished plant bug management in cotton across the Mid-South.

Tarnished plant bugs may cause damage to cotton in all growth stages, but most economic damage occurs from the first square through to the fourth or fifth week of bloom [11,12]. Early in the squaring season, migratory adult plant bugs primarily feed on developing “pinhead” squares. This feeding causes squares to abort, or abscise, from the plant, leading to delayed maturity and often decreased yield if greater than 20% of the squares are aborted [11,13,14]. In addition to feeding on pinhead squares, adult tarnished plant bugs also lay eggs that hatch into nymphs over the next 7 to 10 days. Tarnished plant bug nymphs prefer to feed on medium to large squares but may also feed in blooms or on small bolls [15]. Although cotton plants do not often abort larger squares after tarnished plant bugs have fed on them, the developing anthers inside the square are often damaged, leading to “dirty” blooms that do not pollinate properly [16]. Large populations of tarnished plant bug nymphs lead to substantial yield losses when not controlled. In addition to resistance, insecticide coverage is another factor that plays into control failures. Often, tarnished plant bug nymphs are hidden inside the square bracts and are protected from exposure to insecticides.

The distribution of tarnished plant bugs in cotton following an insecticide spray is a topic that needs further research. Fye reported that approximately 85% of insects recorded in untreated cotton plants were found in the upper 2/3rds (0.61 m) of the plant canopy [17]. Snodgrass found that 75% of adult and nymph tarnished plant bugs were found on the upper six nodes of the main stem in untreated cotton [18]. In the same study, a strong preference for fruiting structures by nymphs was observed once squares (flower buds) were present. Adults were found in high numbers on vegetative structures during the first 3 weeks of squaring, then moving to reproductive structures toward the end of the season. In a study of *Lygus Hesperus*, Knight showed this pest to be found mainly on the upper five to seven nodes [19]. This study also reported that nymphs preferred to feed on squares and adults on bolls. Pack and Tugwell [15] showed that tarnished plant bugs preferred pinhead squares over larger squares or bolls.

The impact of insecticide classes on the behavior and distribution of tarnished plant bugs in cotton is also important. Fontenot reported a significantly higher proportion of tarnished plant bug nymphs on squares than on white flowers or bolls in acephate-treated cotton at various sampling periods ranging from 24 to 120 h after treatment [20]. The only difference between the distribution of tarnished plant bugs on acephate-treated and untreated cotton was observed 24 h after treatment. A significantly lower proportion of nymphs was observed on acephate-treated squares than on untreated squares. Fontenot also studied the vertical distribution of nymphs on acephate-treated and untreated plants [20]. A significantly higher proportion of nymphs was observed in the upper and middle thirds than the lower thirds of untreated plants, while a greater percentage of nymphs were observed in the middle third of the canopy after treatment with acephate and in the upper third of the canopy in the untreated cotton. The authors speculated that this could be because the highest mortality rate occurred in the upper third of the canopy directly after application. As the lethal residual decays, the upper level could be re-infested by nymphs migrating up the plant or by newly hatched nymphs [20]. This is consistent with Lawson et al., who reported significantly higher pesticide residues on the leaves in the upper portion of the canopy [21]. The behavioral response of tarnished plant bug to other insecticides has not been investigated. Currently, there is little information available about the behavioral response of tarnished plant bug to insecticides used for their control. This information may be important for explaining the spatial and temporal distribution of tarnished plant bug in cotton that has been treated with various insecticides. The objective of this experiment was to determine the behavioral response of tarnished plant bug to several classes of insecticides in the laboratory.

## 2. Materials and Methods

Experiments were conducted at the Clay Lyle Entomology Complex in Starkville, MS, in 2014 and 2015 to determine the response of tarnished plant bug nymphs to selected insecticides representing the organophosphate, carbamate, pyrethroid, neonicotinoid, insect growth regulator, sulfoxamine, and pyridine carboxamide classes. These experiments were conducted using third-instar nymphs from a laboratory-reared colony at Mississippi State University, originally reared in 2005. The initial colony was collected from weedy hosts in Mississippi and was supplemented periodically via feral populations collected in Mississippi over time. Prior to initiation of the study, nymphs were reared on semi-solid oligidic diet packs that contained blended whole chicken eggs, sterile water, sugar, Brewer’s yeast, 50% honey solution, and a 10% acetic acid solution [22] that also included fumagillin at 33.6 ppm [23]. The colony was maintained on a 14:10 L:D cycle at 27 °C and 70% humidity. Twenty nymphs were aspirated into 1.5 mL scintillation tubes. Green beans (*Phaseolus vulgaris* L.) were washed in a 5% sodium hypochlorite bath for 5 min, rinsed with clean water, and placed under a vent hood to dry. Green bean pods were then cut into 1.27 cm long sections and submerged into mixtures of insecticides for 3 seconds using a stainless steel mesh strainer. After treatment, the green bean pieces were placed on paper towels and allowed to air dry. Each insecticide was mixed in separate 4.64 L stainless steel sprayer tanks with concentrations calculated based on 112 L per ha spray volume.

The insecticides and mix rates tested included imidacloprid (Admire^®^ Pro, Bayer Crop Science, Raleigh, NC, USA) at 7.4 mL a.i. per ha, thiamethoxam (Centric^®^, Syngenta Crop Protection, Inc., Greensboro, NC, USA) at 13.6 g a.i. per ha, sulfoxaflor (Transform WG™, Dow AgroSciences, Indianapolis, IN, USA) at 8.6 g a.i. per ha, acephate (Orthene 90S WSP, Valent USA, Walnut Creek, CA, USA) at 148.6 g a.i. per ha, oxamyl (Vydate^®^ C-LV, DuPont Crop Protection, Wilmington, DE, USA) at 52.3 g a.i. per ha, bifenthrin (Brigade^®^ 2EC, FMC Corporation, Princeton, NJ, USA) at 18.3 mL a.i. per ha, permethrin (Ambush^®^, AgNova Technologies, Box Hill North Vic, Australia) at 36.7 mL a.i. per ha, flonicamid (Carbine™ 50WG, FMC Corporation, Wilmington, DE, USA) at 16.3 g a.i. per ha, novaluron (Diamond^®^ 0.83EC, ADAMA USA, Raleigh, NC, USA) at 57 mL a.i. per ha, and water for an untreated control.

The assay arenas consisted of individual 245 mm square polystyrene bioassay dishes (Corning™ Product Number 431272, Corning, NY, USA) that were divided into four quadrants. Five green bean pieces were placed into each quadrant for a total of 20 green bean sections per dish. Two of the quadrants contained treated green beans, and the other two quadrants contained untreated green beans. Green bean pieces were randomly assigned (treated or untreated) to each quadrant within each replication. Twenty green bean sections were chosen to allow for a 1:1 ratio of green bean host substrate to tarnished plant bugs to ensure that there were enough green beans to host the tarnished plant bugs and minimize crowding. Assays were conducted a total of seven times over the 2-year period, but not all insecticides were evaluated at every assay. Insecticides were grouped based on IRAC MoA chemical sub-group or exemplifying active ingredient (Table 1). In each assay, treatments were replicated four times, with one dish per replication for a total of four dishes per treatment. Tarnished plant bugs were emptied from the scintillation tubes in the center of the dishes, attempting to avoid placing them closer to the treated or untreated green beans. The dishes were wrapped in parafilm to ensure no tarnished plant bugs were able to escape. The dishes were checked at intervals of 1 h, 4 h, 8 h, and 24 h, and the location (treated or untreated green bean sections and/or quadrant) of dead and live tarnished plant bugs was recorded. Tarnished plant bugs that were found dead were not included in the analysis because they did not have the option to move throughout the area.

## 3. Data Analysis

The proportion of live tarnished plant bug nymphs on treated green beans (or their quadrants) was calculated at each rating for each insecticide. Data at the 24 h rating was analyzed with a general linear mixed model analysis of variance for repeated measures [24]. Insecticide class was designated as a fixed effect in the model; time was the repeated term. Test and replication nested within the test were designated as random effects in the model. Degrees of freedom were estimated using the Kenward-Roger method [25]. Means were estimated using LSMEANS and separated based on Fisher’s protected least significant difference (α = 0.05). Additionally, the relationship between the proportions of tarnished plant bug nymphs on treated green bean pieces over time was analyzed with regression analysis for each insecticide and insecticide class.

## 4. Results

**Figure 1:**
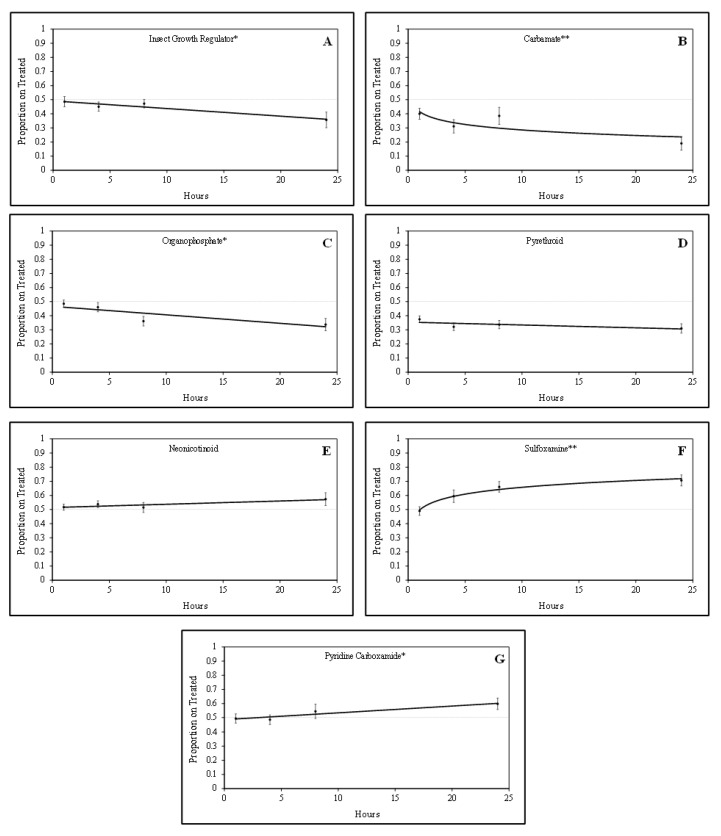
Relationship between the proportion of tarnished plant bug nymphs on treated green beans over time for various insecticide groups based on laboratory bioassays: (**A**) insect growth regulator; (**B**) carbamate; (**C**) organophosphate; (**D**) pyrethroid; (**E**) neonicotinoid; (**F**) sulfoxamine; (**G**) pyridine carboxamide; * significant linear relationship; ** significant quadratic relationship.

Tarnished plant bug nymphs were attracted to green bean pieces treated with sulfoxamine and pyridine carboxamide insecticides. There was a positive quadratic relationship (F = 5.24; df = 1, 61; P = 0.03; R^2^ = 0.23) for the proportion of tarnished plant bug nymphs on sulfoxamine-treated green bean pieces over time (Figure 1). In contrast, there was a positive linear relationship (F = 4.52; df = 1, 61; *p* = 0.04; R^2^ = 0.07) for the proportion of tarnished plant bug nymphs on pyridine carboxamide-treated green bean pieces over time. For the sulfoxamine insecticide, attraction to the treated green bean pieces occurred rapidly, and attraction to the pyridine carboxamide occurred gradually over time (Figure 1).

Tarnished plant bug nymphs appeared to avoid green bean pieces treated with IGR, organophosphate, and carbamate insecticides. There were significant negative linear relationships for the IGR insecticide (F = 6.04; df = 1, 46; *p* = 0.02; R^2^ = 0.12) and the organophosphate insecticides (F = 8.92; df = 1, 62; *p* < 0.01; R^2^ = 0.13) for the proportion of tarnished plant bug nymphs observed on treated green bean pieces over time (Figure 1). There was a significant negative quadratic relationship (F = 3.06; df = 1, 76; *p* = 0.08; R^2^ = 0.11) for the proportion of tarnished plant bug nymphs observed on treated green bean pieces for the carbamate insecticides. Although some of the R^2^ values are relatively low, the regressions are significant, so they are still meaningful.

No relationship (F = 1.56; df = 1, 155; *p* = 0.21; R^2^ = 0.01) was observed for the proportion of tarnished plant bug nymphs observed on neonicotinoid-treated green bean pieces over time in these studies (Figure 1). Similar to neonicotinoids, there was no relationship (F = 1.58; df = 1, 142; *p* = 0.21; R^2^ = 0.01) for the proportion of tarnished plant bug nymphs on pyrethroid-treated green bean pieces over time (Figure 1). In laboratory studies, tarnished plant bug nymphs appeared to rapidly avoid carbamates and slowly avoid the IGR and organophosphate insecticides over time. As treatments were rated at the 1-, 4-, and 8-h intervals, it was apparent that the tarnished plant bug nymphs were gaining a stronger attraction or avoidance to each respective treatment leading up to the 24 h rating (Table 2), when the tarnished plant bug nymphs had ample time to make behavioral responses.

## 5. Discussion

In the case of the neonicotinoid insecticides, there appeared to be a slight increase over time in the proportion of tarnished plant bug nymphs on treated green beans in the laboratory. The attraction to neonicotinoid-treated sucrose solutions has been shown with honeybees (*Apis melifera* (L.)) and bumblebees (*Bombus terrestris* (L.)) [26]. The active ingredients used in the study by Kessler et al. [26] were the two used in this study: imidacloprid and thiamethoxam. However, we did not observe a behavioral response of tarnished plant bug nymphs to neonicotinoids within the 24-h period evaluated. It is not known what may have happened after the 24-h period, and we cannot conclude that there is no attraction. A study by Fernandes et al. [27] reported that the predator insects *Cycloneda sanguinea*, *Orius insidiosus*, and *Chauliognathus flavipes* were repelled by the presence of neonicotinoid insecticides. This study suggests that different insects and insect types may behave differently in the presence of insecticides, such as neonicotinoids. This could be explained by differences in food source detection by insect pest species and insect predator species. In addition, the response of adult insects may be stronger to weaker stimulants than immature insects because they are less mobile by nature.

Although the relationship was not significant, tarnished plant bug nymphs appeared to avoid pyrethroid insecticides in laboratory trials. At the initial rating interval (1 h), the percentage of tarnished plant bug nymphs on pyrethroid-treated green beans was already less than 40%. By 24 h, this proportion had declined to 31%. In a field study observing the behavioral response of honeybees to insecticides, the presence of permethrin caused honeybees to avoid entering treated fields [28]. Similarly, Fernandes et al. found that predator insects were repelled by pyrethrins (IRAC Group 3A) [27]. The results found by those studies were similar to results with the tarnished plant bug nymphs in this study, which observed a non-significant trend for third-instar nymphs to avoid green beans treated with permethrin.

In this study, tarnished plant bug nymphs avoided insect growth regulator, organophosphate, and carbamate insecticides. Rust et al. reported adult German cockroaches (*Blatella germanica* (L.)) were repelled by organophosphate and carbamate insecticides in Ebling choice box tests in resistant populations [29]. This study was conducted on a laboratory colony of third-instar tarnished plant bug nymphs, which are susceptible to these chemistries. However, tarnished plant bugs have shown the ability to develop resistance to these insecticides in the field [10]. It is possible that the behavioral response (avoidance) by non-resistant populations led to sublethal ingestion of insecticides, leading to the resistance found in field populations in Mississippi. A study found that German cockroaches are repelled by Group 15 insecticides [30], similar to the avoidance found by tarnished plant bugs in this study. Currently, no resistance has been reported for tarnished plant bugs to Group 15 insecticides. The studied active ingredient here, novaluron, is a key insecticide used to manage immature populations of tarnished plant bugs in cotton. Understanding the behavioral response of tarnished plant bugs when exposed to this chemical can help to add knowledge for insecticide resistance management programs. Tarnished plant bug nymphs were attracted to sulfoxaflor (IRAC Group 4C) and flonicamid (IRAC Group 29), two relatively new insecticide chemistries for tarnished plant bug control. Currently, no field-evolved resistance has been documented to these insecticides for tarnished plant bugs. The attraction behavior in response to these insecticides can potentially help delay resistance development by increasing the amount of chemicals ingested if tarnished plant bugs are attracted to these compounds.

With the widespread occurrence of resistance to multiple classes of insecticides [2,5,8,9,10,12]), understanding the behavioral response of tarnished plant bug to those classes of insecticide may improve our understanding of control and control failures. Spraying insecticides that invoke behavioral responses could help improve efficacy or lead to control issues if there are areas that are not treated (i.e., dense canopy). Using insecticides that tarnished plant bug tends to avoid could cause them to potentially move out of protected structures of the plant, such as square bracts, and put them in direct contact with the insecticide. Similarly, using insecticides that tend to attract tarnished plant bug could cause them to move into better contact with the insecticide. However, if tarnished plant bugs avoid insecticides, that could cause them to ingest sublethal levels of insecticide, leading to resistance. Obviously, if tarnished plant bug does have an avoidance behavior to insecticides, this could also cause them to flee to lower parts of the cotton canopy, where there is little to no insecticide coverage, leading to control failures.

The present study only observed the behavioral response of third-instar tarnished plant bug nymphs to classes of insecticides. Studies to observe the response of adult tarnished plant bugs to chemicals used to manage adult populations should also be conducted. A study by Graham et al. evaluating tarnished plant bug behavioral responses to a new Bt toxin under development (Cry51Aa2) found that while first- and third-instar tarnished plant bug nymphs did not have an observable response, adult tarnished plant bugs avoided Cry51Aa2 in diet-pack assays and in choice studies with cotton squares [31]. The avoidance of food sources led directly to reduced egg-lay [31], thus minimizing the number of tarnished plant bug nymphs that would hatch out over the coming week. If adult tarnished plant bugs are attracted to, or avoid, chemical insecticides used for controls on migrating adult populations, there is potential to affect future nymphal populations within the field. In this study, we did not consider the role of certain effects, such as insecticide odor or residual dried insecticide film left on green bean pieces after dipping into the insecticide mixture had on the tarnished plant bug responses. Further research needs to be conducted to better understand the behavioral responses of the tarnished plant bug to chemical insecticides.

## Figures and Tables

**Table 1:** List of insecticide treatments used for the impact of tarnished plant bug behavior in laboratory bioassays in 2014–2015.

Insecticide	Class	Tests	Replications per Test
Oxamyl	1A	4	4
Dicrotophos	1B	1	4
Acephate	1B	4	4
Permethrin	3A	3	4
Bifenthrin	3A	6	4
Imidacloprid	4A	3	4
Thiamethoxam	4A	7	4
Sulfoxaflor	4C	4	4
Flonicamid	29	4	4
Novaluron	15	3	4

**Table 2:** Proportion of tarnished plant bug nymphs attracted to treated green beans 24 h after treatment in laboratory bioassays during 2014 and 2015.

Treatment	Proportion Attracted *	Proportion Dead at 24 H **
Sulfloxamines	0.71 (0.04) a	0.09
Pyridine Carboxamides	0.60 (0.04) ab	0.11
Neonicotinoids	0.57 (0.05) ab	0.16
Insect Growth Regulators	0.36 (0.05) bc	0.03
Organophosphates	0.34 (0.04) bc	0.19
Pyrethroids	0.31 (0.03) bc	0.14
Carbamates	0.19 (0.05) c	0.31

* Means within the column that are followed by the same number are not different according to Fisher’s Protected LSD (alpha = 0.05). ** Total proportion of dead nymphs at 24-h rating.

## Data Availability

Data available on request.
